# Compliance and Adherence to Occlusal Splint Therapy and Physiotherapy in Temporomandibular Disorders: A Retrospective Cohort Study Using Narrative‐Derived Global Impression of Change

**DOI:** 10.1155/ijod/3585953

**Published:** 2026-11-01

**Authors:** Nadav Grinberg, Ofek Shpigler, Oren Peleg, Clariel Ianculovichi, Shlomi Kleinmann, Reema Mahmoud, Ayelet Zlotogorski-Hurvitz, Gilad Wasserman

**Affiliations:** ^1^ Department of Oral and Maxillofacial Surgery Tel-Aviv Sourasky Medical Center Tel Aviv 64239 Israel tau.ac.il; ^2^ Goldschleger School of Dental Medicine Faculty of Medical and Health Sciences Tel Aviv University Tel Aviv Israel tau.ac.il; ^3^ Oral Medicine Unit Tel Aviv Sourasky University Medical Center Tel Aviv Israel

**Keywords:** occlusal splints, patient-reported outcome measures, physical therapy modalities, temporomandibular disorders, treatment adherence and compliance

## Abstract

**Background:**

Occlusal splints and physiotherapy are the mainstays of conservative management for temporomandibular disorders (TMDs), but how often patients follow these recommendations, and what follows when they do not, is poorly quantified.

**Methods:**

From 3317 patients with a TMD diagnosis and at least two clinic visits, 800 records were randomly selected and screened; 273 met inclusion criteria and contributed 443 recommendation events. Adherence was classified by initiation and persistence. A proxy Patient Global Impression of Change (PGIC) score was derived from the follow‐up narrative under a predefined rule. The primary outcome was PGIC ≤2.

**Results:**

Persistence after initiation was 70% for splints, 88% for self‐administered and 90% for professional physiotherapy. Splint losses were characteristically after acquisition (40 of 133 acquirers discontinued), whereas professional physiotherapy failed mostly before initiation (66 of 174 never attended). Among 66 patients recommended both physiotherapy types, the home programme was performed far more often (20 versus 1 discordant pairs, *p* < 0.001). Of 133 obtaining a splint, 122 (92%) received the prescribed appliance; deviation was concentrated in community‐fabricated splints (odds ratio [OR] 6.37, 95% confidence interval [CI] 1.60–25.39). Adherence was associated with improvement in every modality (splint OR 10.35, 95% CI 4.31–24.82; professional physiotherapy OR 5.05, 1.97–12.92; self‐administered physiotherapy 37% versus 0%, *p* < 0.001; pooled OR 8.24, 4.05–16.79); response rose from 4% to 45% across adherence strata. Apart from a joint‐only diagnosis, no baseline characteristic predicted response after adjustment (adherence adjusted OR [aOR] 12.00, 5.40–26.67). Restricting the contrast to never‐initiated versus adherent events, which excludes response‐dependent discontinuation, gave OR 9.11 (4.26–19.50).

**Conclusions:**

About 40% of conservative TMD recommendations were not followed, and non‐adherence was associated with a substantially lower probability of meaningful improvement, although residual confounding, including a healthy‐adherer effect, cannot be excluded. Non‐initiation and discontinuation are distinct problems with distinct causes.

## 1. Introduction

Temporomandibular disorders (TMDs) are among the most common causes of chronic orofacial pain [1, 2]. International guidance places conservative, reversible measures first [3, 4] and in most specialist units, this means a stabilisation splint [5, 6] and physiotherapy, delivered either as a self‐administered home programme or by a physiotherapist [7, 8].

The efficacy of both is supported by randomised evidence [5, 7, 9], but efficacy trials measure what happens when patients use a treatment. They say little about what happens in a clinic, where a recommendation is made, and the patient may or may not obtain the appliance, attend physiotherapy, or persist. Adherence is the step between an evidence‐based recommendation and any prospect of benefit, and across chronic disease it is routinely assumed rather than measured [10, 11]. Where it has been examined in TMD, reported splint wear varies widely and is seldom separated from the initial acquisition [12, 13]. In a specialty clinic cohort monitored by electronic diaries, median compliance ranged from under 10% to over 90% depending on the treatment recommended [14] and in a recent multicentre questionnaire study, two‐thirds of patients reported high splint adherence four to eight weeks after insertion [15].

Three gaps motivated this study. First, adherence has rarely been quantified separately for each modality within one cohort, so it is unknown whether splints and physiotherapy fail at similar rates or for similar reasons. Second, where a recommendation specifies a particular appliance, it is unknown how often patients receive that appliance rather than another device, a question with clinical consequences because soft and hard appliances are not therapeutically equivalent [16, 17]. Third, the relationship between adherence and patient‐reported outcome has not been characterised with a graded exposure that would permit a dose–response analysis.

A practical obstacle to answering these questions retrospectively is the outcome measure. Validated patient‐reported instruments are seldom administered in routine care. Clinical notes nevertheless contain a narrative account of how the patient reported feeling at follow‐up, and we reasoned that this could be converted under an explicit rule into a proxy of the Patient Global Impression of Change (PGIC), a validated single‐item instrument endorsed by the Initiative on Methods, Measurement, and Pain Assessment in Clinical Trials (IMMPACT) whose response options are themselves plain‐language descriptions of global change [18–21].

We therefore set out to quantify initiation of and persistence with each modality; to determine whether the appliance recommended was the appliance received and whether this depended on where it was fabricated; to characterise documented reasons for non‐adherence; and to relate adherence to a proxy patient‐reported outcome derived from the clinical narrative.

## 2. Materials and Methods

### 2.1. Design, Setting and Participants

This was a single‐centre retrospective cohort study at the oral medicine unit, reported in accordance with the STROBE statement [22]. Patients were identified through MDClone, the institutional clinical data‐exploration platform, by querying for a TMD diagnosis (ICD‐9 524.60) with at least two clinic visits between 1 August 2018 and 30 March 2025, which yielded 3317 patients. The two‐visit criterion was part of the query definition, so patients with a single visit were never extracted, and their number could not be determined. The exported list was randomised in Microsoft Excel, and the first 800 records were selected for manual screening; the draw was not seed‐documented and is therefore not exactly reproducible. The sample of 800 was set by the feasibility of manual narrative abstraction by a single trained assessor; no formal sample‐size calculation was performed. Eligible patients had a TMD diagnosed by the treating clinician according to Axis I of the Diagnostic Criteria for TMDs [1], at least one documented recommendation for an occlusal splint, self‐administered physiotherapy or professional physiotherapy and a follow‐up assessment documented in sufficient narrative detail to permit outcome derivation. Patients lacking any of these were excluded. Two hundred and seventy‐three patients met all criteria and contributed 443 recommendation events (Figure 1). Of the 527 records excluded, 212 had no follow‐up appointment, 180 had no splint or physiotherapy recommendation, 117 contained insufficient text for data extraction and 18 had a diagnosis that was not clearly TMD. Diagnoses were recorded in up to three fields, so a patient with both muscular and articular involvement could carry both labels; muscular categories comprised local myalgia, myalgia, myofascial pain and myofascial pain with referral, and articular categories comprised disc displacement with and without reduction, arthralgia and degenerative joint disease.

**Figure 1:**
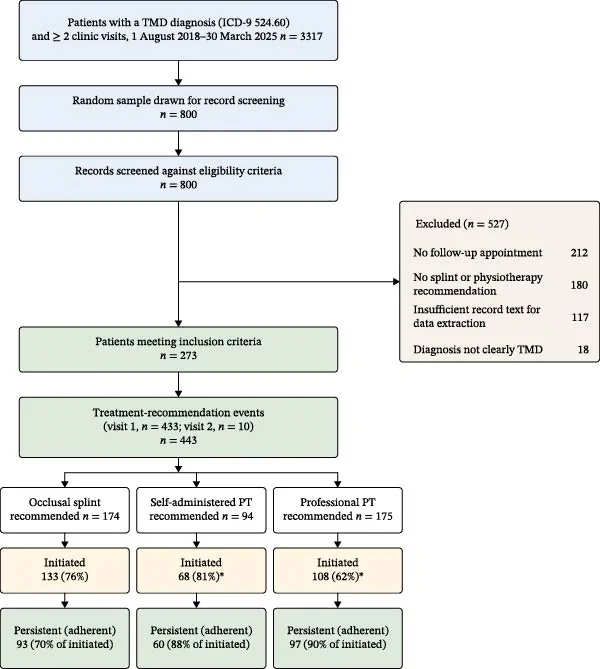
Study flow diagram.  ^∗^Initiation status not determinable for 10 self‐administered and 1 professional physiotherapy event; percentages use determinable events (84 and 174). Adherent = initiated and not discontinued by the outcome visit.

### 2.2. Treatment Recommendations

Every splint recommendation was for the same appliance: a full‐arch rigid acrylic Michigan‐type stabilisation splint for nocturnal wear, usually on the mandibular arch [5, 23]. No other appliance type was recommended for any patient. Because the prescription was constant, any variation in the appliance received represents a departure from the recommendation rather than a difference in clinical intent. Splints were fabricated within the unit or obtained from a community practitioner at the patient’s choice. Self‐administered physiotherapy was a standardised home programme of jaw exercises and self‐management instruction taught at the recommending visit [8]. Professional physiotherapy was a referral for supervised treatment, requiring the patient to obtain an appointment and attend externally [7].

### 2.3. Outcome Derivation: Narrative‐Based Proxy PGIC

The PGIC is a single‐item seven‐point instrument on which the patient rates the overall change since starting treatment, from 1 (very much improved) through 4 (no change) to 7 (very much worse). It is endorsed by IMMPACT as a core outcome domain for chronic pain and has established interpretive thresholds [18, 19, 21]. It was not administered prospectively in this clinic.

The instrument lends itself to retrospective derivation because its response options are plain‐language descriptions of global change rather than numerical anchors requiring calibration. A note recording that a patient reports being much better since starting treatment contains, in substance, the information a PGIC response captures; the task is to map existing natural language content onto a fixed set of natural language categories under an explicit rule. The resulting score is a proxy: it reflects the patient’s report as documented by the treating clinician, not a rating made by the patient on the instrument itself and it is referred to throughout as the derived PGIC.

For each recommendation event, the free‐text follow‐up narrative was extracted from the medical record together with any documented change in symptoms, function or medication use. The outcome was taken from the first follow‐up visit after the recommendation; later visits were not used to reassign it. Because the PGIC rates global change, one score was assigned per patient visit and applied to every modality recommended at that visit. All derivations were performed by a single assessor (Ofek Shpigler), who underwent training and calibration against the mapping rule on a pilot set of records before extraction began. Scores were assigned from 1 to 7 according to a mapping rule agreed upon before extraction. Adherence status and outcome were abstracted from the same clinical narrative in a single pass, and the text was not masked before scoring; blinding of the assessor to adherence was therefore not achieved, and the resulting potential for correlated measurement error is addressed in the limitations. The rule anchored each score to the standard PGIC descriptor: unambiguous resolution or near‐resolution to 1; clear substantial improvement to 2; partial or equivocal improvement, or improvement in one domain only, to 3; explicit absence of change, or continuing symptoms recorded without qualification, to 4; and the corresponding descriptors for deterioration at 5–7. Narratives insufficient to support any assignment were to be recorded as missing rather than defaulted to no change since defaulting would bias the outcome toward the null in the direction that inflates an apparent adherence effect. In practice, this rule was never invoked because records with insufficient narrative had already been excluded at eligibility (*n* = 117); eligibility is therefore outcome‐dependent, a point returned to in the limitations.

Because derivation was performed by one assessor and duplicate coding was not feasible, no inter‐rater reliability coefficient could be calculated. As an external check, the record separately contained a three‐level clinician‐assigned treatment effect rating (none, slight, major) entered at the follow‐up consultation by the treating clinician and not derived from the narrative, allowing the derived scores to be compared against a contemporaneous independent judgement. This comparison tests internal consistency with the clinician’s judgement; it is not a validation against a patient‐administered PGIC.

### 2.4. Initiation, Persistence and Adherence

Adherence was described using the ABC taxonomy of Vrijens et al. [11], which distinguishes initiation, when the patient starts the recommended treatment; implementation, the extent to which actual use matches the prescribed regimen; and persistence, continuation of treatment over time, which ends in discontinuation. Initiation and persistence were recorded. Implementation, such as the number of nights per week a splint was worn or how often home exercises were performed, was not documented in a recoverable form and was not analysed. A splint recommendation was initiated when the appliance was obtained and a physiotherapy recommendation when the patient reported performing the home programme or attending physiotherapy; an initiated event was persistent when no discontinuation was documented by the outcome visit. An event was classified as adherent when it was both initiated and persistent and as non‐adherent when it was never initiated or was discontinued; this binary exposure is used in all outcome analyses. The discontinuation field applied to physiotherapy generally and was attributed to whichever modality the patient had actually initiated; no patient initiated both physiotherapy types and then discontinued, so attribution was unambiguous throughout. At the patient level, three summary measures were derived: fully adherent, meaning adherent to every modality recommended; any adherence; and adherence proportion, the number of modalities adhered to divided by the number recommended, used as a continuous variable to test for dose–response. Prescription fidelity was assessed among patients who obtained a splint by comparing the appliance received against the uniformly recommended rigid Michigan appliance.

### 2.5. Analysis

Because the outcome was assessed once per visit, while most patients received more than one modality (132 patients one, 122 two, 19 all three), analyses were conducted at the recommendation‐event level, where each patient‐visit‐modality combination is one observation, and at the patient level, where each patient contributes one observation and clustering is eliminated. All recommendation visits were included rather than the first visit only because 10 events arose from a second recommendation visit; restriction to first‐visit recommendations was examined as a sensitivity analysis. A fixed follow‐up time point could not be imposed retrospectively, because follow‐up was scheduled clinically, so the first follow‐up visit after each recommendation was used and the interval to it was analysed as described below. Continuous variables are reported as mean with standard deviation when approximately symmetric (age, maximum interincisal opening) and as median with interquartile range when skewed (follow‐up interval); categorical variables as counts and percentages.

Prespecified covariates were age, sex, symptom duration in bands of ≤3, > 3–6, > 6–12 and >12 months with boundaries fixed before the outcome was examined, bruxism, operationalised during the study period according to the 2018 international consensus [24] and reported here using the nomenclature of the subsequently published Standardised Tool for the Assessment of Bruxism as clinically probable [25], psychiatric comorbidity, other chronic pain, prior splint therapy, current splint use at the recommendation visit, number of modalities recommended, and diagnostic pattern derived from all three diagnosis fields as muscle only, joint only or mixed. Comorbidities were abstracted as binary fields and recorded as present when the condition appeared as a listed diagnosis or a medication prescribed for it was listed in the record; no validated questionnaire scores were available. The anxiety and depression fields were coded identically in all 273 patients (51 positive) and could not be separated, so they were combined as a single psychiatric comorbidity indicator.

Modality‐specific associations were estimated by the Fisher exact test with odds ratios (ORs) and 95% confidence intervals (CIs) from the log‐odds and its standard error, applying the Haldane–Anscombe correction of 0.5 for estimation only where a zero cell occurred [26]; such estimates indicate direction rather than magnitude and are flagged. All CIs are Wald intervals unless stated otherwise, and all *p*‐values for two‐by‐two comparisons are from Fisher exact tests. Pooled estimates used generalised estimating equations (GEE) with a binomial family, logit link, patient as cluster and robust standard errors [27]. An independence working correlation was specified: because a single outcome is recorded per visit and applies to every modality recommended at that visit, the outcome is duplicated within clusters by design, and an exchangeable structure absorbed this artefactual correlation (estimated *α* = 0.80) and materially shifted the point estimate. Under independence, the marginal estimate is recovered unchanged from the crude value, as theory requires, while the robust standard error retains the correction for clustering. The trend across adherence strata was tested by the Cochran–Armitage test. Patient‐level associations used logistic regression assessed by the *C*‐statistic, McFadden pseudo‐*R* ^2^, the Hosmer–Lemeshow test and variance inflation factors [28], with the events‐per‐variable ratio reported [29]; adjusted ORs (aORs) are reported from this model. Because this ratio was below 10, the multivariable model was refitted using Firth penalised likelihood [30]. Covariate entry into the multivariable model was prespecified and was not based on univariable *p*‐values.

Prespecified sensitivity analyses repeated the comparison at PGIC ≤3 and modelled the full seven‐level scale by proportional‐odds ordinal regression, avoiding the information loss of dichotomisation [31]. The proportional‐odds assumption was tested formally by a likelihood‐ratio test comparing the proportional‐odds model with a multinomial logistic model, PGIC 5 and 6 being pooled because of sparse cells, and by a Brant‐type Wald test of equal coefficients across the cumulative binary models fitted at each cut‐point [32]; equality of coefficients across the three improvement cut‐points (PGIC ≤1, ≤ 2 and ≤3) was tested separately.

The interval from recommendation to the outcome visit was calculated for every event and compared across adherence categories by the Kruskal–Wallis test. Its influence on the adherence–outcome association was examined by adding it as a covariate to the patient‐level multivariable model and by restricting that model to intervals of 1–12 months.

The primary exposure, primary outcome, covariate set, symptom duration bands, the PGIC ≤3 and ordinal sensitivity analyses and the domains for reasons for non‐adherence were specified before outcome data were examined. The following analyses were added afterwards, in response to internal or external review or because the data suggested them, and are post hoc: Firth penalised regression; the contrast restricted to never‐initiated versus adherent events; the analysis restricted to visits at which a single modality was recommended; the analysis restricted to first‐visit recommendations; the formal proportional‐odds tests; the follow‐up interval analyses; and the comparisons of response by diagnostic pattern and by fabrication source. Post hoc results are labelled as such and interpreted as exploratory.

Analyses were complete case, and no values were imputed. Symptom duration was missing for 3 patients and pre‐treatment maximum interincisal opening for 22. Initiation or persistence could not be determined for 11 events in 11 patients (10 self‐administered physiotherapy and 1 professional physiotherapy); these events were excluded from the adherence analyses and these patients from the patient‐level analysis (*n* = 262). For reasons for non‐adherence, a blank field and an explicit entry of no reason given were both treated as the absence of a substantive reason; the two were not distinguished in any analysis.

The principal potential sources of bias and the steps taken were selection bias from eligibility criteria requiring a follow‐up visit and adequate documentation, which could not be corrected with the available data and is discussed below; information bias from derivation of the outcome by a single assessor who was not blinded to adherence, mitigated by a predefined mapping rule, assessor calibration and comparison with an independent clinician rating; confounding, including confounding by indication and the healthy‐adherer effect, addressed by prespecified multivariable adjustment and a within‐patient comparison; and reverse causation through response‐dependent discontinuation, addressed by the never‐initiated versus adherent contrast.

Reasons for non‐adherence were classified before frequencies were examined into structural, clinical, communication, attitudinal and treatment‐not‐required domains. Two‐sided tests were used with a threshold of 0.05; no multiplicity correction was applied because the primary hypothesis concerned a single prespecified exposure and the remaining tests are covariate screens intended to demonstrate the absence of confounding, with any incidental nominal association identified as such. Analyses used Python 3.12 (pandas, NumPy, SciPy, statsmodels), with key contingency analyses replicated in IBM SPSS Statistics.

### 2.6. Ethics

The study was approved by the institutional Helsinki committee (Protocol 0339‐25‐TLV) with a waiver of individual consent for the retrospective use of existing records. All data were handled de‐identified.

## 3. Results

### 3.1. Cohort

Of 800 randomly selected records screened, 273 patients met inclusion criteria and contributed 443 recommendation events (Figure 1). A second recommendation visit, at which further treatment was recommended, was recorded for six patients, contributing 10 additional events; none had a third. Mean age was 47.3 years (SD 18.9; range 16.7–89.9), and 199 patients (73%) were female. The median interval from recommendation to the outcome visit was 122 days (IQR 91–198; range 7–1780). Ninety‐nine patients had muscle involvement only, 108 had joint involvement only and 66 had both. Mean pre‐treatment maximum interincisal opening was 46.2 mm and was greatest in muscle‐only disease (48.4 mm versus 45.0 mm in both other groups; Kruskal–Wallis *p* = 0.029), supporting the validity of the classification (Table 1).

**Table 1:** Cohort characteristics (*n* = 273).

Characteristic	Value	*n* with data
Demography		
Age, years—mean (SD)	47.3 (18.9)	273
Female—*n* (%)	199 (73)	273
Married—*n* (%)	114 (42)	273
Disease		
Symptom duration ≤3 months—*n* (%)	58 (21)	270
Symptom duration >3–6 months—*n* (%)	81 (30)	270
Symptom duration >6–12 months—*n* (%)	45 (17)	270
Symptom duration >12 months—*n* (%)	86 (32)	270
Pre‐treatment MIO, mm—mean (SD)	46.2 (9.5)	251
Diagnostic pattern (all diagnosis fields)—*n* (%)		
Joint involvement only	108 (40)	273
Muscle involvement only	99 (36)	273
Mixed muscle and joint	66 (24)	273
Comorbidity and history—*n* (%)		
Bruxism, clinically probable	58 (21)	273
Anxiety or depression (listed diagnosis or medication)	51 (19)	273
Other chronic pain	58 (21)	273
Central sensitisation marker	74 (27)	273
Current psychiatric medication	54 (20)	273
Current chronic‐pain medication	61 (22)	273
Prior splint therapy	68 (25)	273
Wearing a splint at recommendation	47 (17)	273
Prior physiotherapy	28 (10)	273
Recommendations at index visit—*n* (%) of patients		
Professional physiotherapy	171 (63)	273
Occlusal splint (rigid Michigan)	169 (62)	273
Self‐administered physiotherapy	93 (34)	273
One/two/three modalities	132/122/19	273
Outcome—*n* (%)		
PGIC ≤2 (clinically meaningful improvement)	75 (27)	273
PGIC ≤3 (any improvement)	145 (53)	273
Fully adherent to all recommendations	135 (52)	262
Recommendation‐to‐outcome interval, days—median (IQR)	122 (91–198)	443 events

*Note:* Recommendation counts are patients receiving each modality at the index visit and sum to 433 index‐visit recommendations (132 patients received one modality, 122 two and 19 three); a further 10 recommendations were made at second visits, giving 443 events in total. Comorbidities were recorded as present when listed as a diagnosis or when a medication for the condition was listed; anxiety and depression were coded identically in every patient and are shown combined.

Abbreviations: MIO, maximum interincisal opening; PGIC, patient global impression of change.

### 3.2. Initiation and Persistence by Modality

The two dominant modalities differed in where in the pathway they were lost. Splint therapy was initiated in 133 of 174 recommendations (76%); of these acquirers, 40 subsequently discontinued, leaving 93 persistent events, so that persistence among those who obtained an appliance was 70%. Counting never‐initiated and discontinued events together, 93 of 174 splint recommendations (53%) were adherent. Splint losses were thus divided almost equally between non‐initiation (41) and discontinuation (40), but discontinuation among acquirers (30%) was roughly three times as frequent as that with either physiotherapy modality. Professional physiotherapy showed the inverse pattern: initiation status was determinable for 174 of the 175 recommendations, of which only 108 (62%) were initiated, 66 patients never attending at all, while just 11 of the 108 who started discontinued, giving persistence of 90% among those who attended but adherence in only 97 of 174 recommendations (56%). Self‐administered physiotherapy had the highest initiation at 68 of 84 determinable events (81%) and persistence among initiators at 88% (60 of 68), giving adherence in 60 of 84 events (71%) (Table 2).

**Table 2:** Initiation of the recommendation and persistence after initiation, by modality (443 recommendation events).

Modality	Recommended	Initiated, *n* (%) of recommended	Never initiated	Status not determinable	Discontinued after initiation	Persistent (adherent), *n* (%) of initiated
Occlusal splint	174	133 (76)	41	0	40	93 (70)
Self‐administered physiotherapy	94	68 (81)	16	10	8	60 (88)
Professional physiotherapy	175	108 (62)	66	1	11	97 (90)
All modalities	443	309 (72)	123	11	59	250 (81)

*Note:* Initiation percentages use as denominator those events in which initiation status could be determined (splint 174, self‐administered physiotherapy 84, professional physiotherapy 174). Persistence percentages express continued use among events that were initiated, and therefore describe persistence rather than overall uptake. In the outcome analyses (Table 3) the non‐adherent group comprises both never‐initiated and discontinued events, so the denominators there differ: of 81 non‐adherent splint events, 41 were never initiated and 40 discontinued; of 24 self‐administered physiotherapy events, 16 and 8; and of 77 professional physiotherapy events, 66 and 11.

**Table 3:** Adherence and clinically meaningful improvement (PGIC ≤2), with sensitivity analyses.

Analysis	Non‐adherent, *n*/*N* (%)	Adherent, *n*/*N* (%)	OR (95% CI)	*p*
Primary analysis—event level: clinically meaningful improvement (PGIC ≤2)				
Occlusal splint	7/81 (9)	46/93 (49)	10.35 (4.31–24.82)	<0.001
Self‐administered physiotherapy	0/24 (0)	22/60 (37)	28.64 (1.66–494.02)^b^	<0.001
Professional physiotherapy	6/77 (8)	29/97 (30)	5.05 (1.97–12.92)	<0.001
Pooled, unadjusted	13/182 (7)	97/250 (39)	8.24 (4.44–15.30)	<0.001
Pooled, GEE (independence, robust SE)	—	—	8.24 (4.05–16.79)	<0.001
Pooled, GEE, first‐visit recommendations only (422 events)^a^	—	—	7.98 (4.29–14.85)	<0.001
Sensitivity analysis 1—event level: any improvement (PGIC ≤3)				
Occlusal splint	26/81 (32)	70/93 (75)	6.44 (3.32–12.49)	<0.001
Self‐administered physiotherapy	5/24 (21)	40/60 (67)	7.60 (2.48–23.34)	<0.001
Professional physiotherapy	19/77 (25)	68/97 (70)	7.16 (3.64–14.08)	<0.001
Pooled, GEE (independence, robust SE)	—	—	6.53 (4.27–9.99)	<0.001
Sensitivity analysis 2—event level: full seven‐level PGIC (proportional odds)^c^				
Occlusal splint	—	—	6.24 (3.41–11.42)	<0.001
Self‐administered physiotherapy	—	—	7.00 (2.56–19.16)	<0.001
Professional physiotherapy	—	—	5.71 (3.06–10.67)	<0.001
Pooled	—	—	5.79 (3.92–8.56)	<0.001
Sensitivity analysis 3 (post hoc)—event level: never‐initiated versus adherent (discontinuers excluded)				
Occlusal splint	3/41 (7)	46/93 (49)	12.40 (3.57–43.00)	<0.001
Self‐administered physiotherapy	0/16 (0)	22/60 (37)	19.29 (1.10–338.05)^b^	0.002
Professional physiotherapy	5/66 (8)	29/97 (30)	5.20 (1.89–14.29)	<0.001
Pooled	8/123 (7)	97/250 (39)	9.11 (4.26–19.50)	<0.001
Sensitivity analysis 4 (post hoc)—event level: visits at which a single modality was recommended				
Occlusal splint	1/30 (3)	30/47 (64)	51.18 (6.39–409.81)	<0.001
Self‐administered physiotherapy	0/2 (0)	4/9 (44)	4.09 (0.15–108.94)^b^	0.491
Professional physiotherapy	0/13 (0)	10/34 (29)	11.57 (0.63–213.23)^b^	0.043
All single‐modality events	1/45 (2)	44/90 (49)	42.09 (5.56–318.79)	<0.001
Patient‐level analysis: one observation per patient (*n* = 262)				
Fully adherent, univariable	—	—	9.64 (4.65–20.00)	<0.001
Adherence proportion, per + 25%	—	—	2.00 (1.59–2.50)	<0.001

*Note:* Sensitivity analyses 1 and 2 were prespecified; analyses 3 and 4 were post hoc. They test whether the primary result depends on the choices made in defining it: analysis 1 relaxes the responder threshold, analysis 2 uses the full seven‐level scale without dichotomisation, analysis 3 removes patients who discontinued, whose non‐adherence could itself be a consequence of failing to improve, and analysis 4 is restricted to visits at which a single modality was recommended, so that the global outcome can be attributed to that modality. Event‐level analyses treat each patient‐visit‐modality combination as one observation and therefore include patients more than once when several modalities were recommended; the patient‐level analysis assigns each patient a single observation, with adherence summarised across all modalities recommended to them, and so contains no repeated observations. The Fisher exact *p*‐value is unaffected.

^a^Post hoc.

^b^Zero cell: the Haldane–Anscombe correction was applied for estimation; the resulting interval is uninformative and the estimate indicates direction only.

^c^The proportional‐odds assumption was formally rejected (likelihood‐ratio *p* = 0.002), the departure being confined to the no‐change/deterioration boundary; ordinal odds ratios express the odds of improving one level on the reversed seven‐point scale, are averages across cut‐points and understate the association with improvement.

Sixty‐six patients were recommended both physiotherapy types at the same visit, allowing a comparison in which patient‐level factors are held constant by design. The home programme was performed by 51 patients, and professional physiotherapy was attended by 33; the status for both was determinable in 64, of whom 31 did both and 12 did neither. Of the 21 discordant pairs, 20 performed the home programme without attending physiotherapy, while one did the reverse (exact binomial *p* < 0.001). The gap between the two physiotherapy modalities is therefore unlikely to reflect stable differences between the patients who receive them.

### 3.3. Prescription Fidelity

A splint was recommended on 174 occasions to 172 patients. Of the 133 patients who obtained an appliance, 122 (92%) received the prescribed rigid device; 11 received another (six soft, three semi‐rigid, two vacuum‐formed). Deviation was strongly concentrated by fabrication source: 3 of 89 splints made within the unit (3%) departed from the prescription, against 8 of 44 obtained in the community (18%). Every soft appliance in the cohort was community‐fabricated. Patients receiving a non‐prescribed appliance achieved the primary outcome in 3 of 11 cases (27%) against 47 of 122 (39%) receiving the prescribed appliance (OR 1.67, 95% CI 0.42–6.62, Fisher exact *p* = 0.535); the direction favours fidelity, but the comparison is underpowered and no conclusion about appliance efficacy follows from it (Table 4).

**Table 4:** Appliance received versus appliance prescribed, by fabrication source (133 patients who obtained a splint).

Fabrication source	Rigid (as prescribed)	Soft	Semi‐rigid	Vacuum‐formed	Deviation, *n* (%)
Within the department	86	0	2	1	3 (3)
Community	36	6	1	1	8 (18)
Total	122	6	3	2	11 (8)

*Note:* All recommendations were for a rigid Michigan‐type appliance. The odds of receiving the prescribed appliance were higher for unit‐fabricated than community‐fabricated splints (OR 6.37, 95% CI 1.60–25.39; Fisher exact *p* = 0.006). Equivalently, the odds of receiving a non‐prescribed appliance were 6.37 times higher for community‐fabricated splints; the two statements describe the same table viewed from opposite margins.

### 3.4. Outcome Distribution and Internal Consistency of the Derived PGIC

Mean‐derived PGIC was 3.15 (SD 1.13). Seventy‐five patients (27%) achieved clinically meaningful improvement (PGIC ≤2) and 145 (53%) reported any improvement (PGIC ≤3); 23 events (5%) in 16 patients were classified as deterioration. A derived score was obtainable for all 443 events, and PGIC 7 was not observed. Correspondence with the independent clinician rating was near‐complete: only three events fell outside a band of correspondence specified before the cross‐tabulation was inspected, giving a Spearman correlation of −0.972, which is negative because the two scales run in opposite directions. This demonstrates that the derivation is internally consistent and auditable rather than that it reproduces a patient‐reported construct, and the correlation is inflated by the collapsed three‐level structure of the comparator. The derived scale nevertheless carries resolution the clinician rating cannot: it separates 42 events scored 1 from 70 scored 2 within the single category of major improvement and identifies the 23 events of deterioration, for which the clinician scale has no corresponding category (Table 5).

**Table 5:** Narrative‐derived PGIC against the independent clinician treatment‐effect rating (443 events).

Clinician rating	PGIC 1	PGIC 2	PGIC 3	PGIC 4	PGIC 5	PGIC 6	Total
Major improvement	42	70	0	0	0	0	112
Slight improvement	0	2	118	0	0	0	120
No improvement	0	0	1	187	21	2	211
Total	42	72	119	187	21	2	443

*Note:* Spearman *ρ* = −0.972. Only three events deviate from a correspondence in which major improvement maps to PGIC 1–2, slight improvement to PGIC 3, and no improvement to PGIC 4 or above. The comparison tests internal consistency with the clinician’s judgement, not validity against a patient‐administered PGIC.

### 3.5. Adherence and Clinically Meaningful Improvement

Adherence was associated with the primary outcome in every modality (Table 3). For splint therapy, 46 of 93 adherent events (49%) achieved PGIC ≤2 against 7 of 81 non‐adherent events (9%), an absolute difference of 40 percentage points. For professional physiotherapy, the corresponding figures were 29 of 97 (30%) and 6 of 77 (8%). For self‐administered physiotherapy no non‐adherent event achieved meaningful improvement (0 of 24) against 22 of 60 adherent events (37%; Fisher exact *p* < 0.001); the OR rests on a zero cell and indicates direction rather than magnitude, and post hoc Firth penalised estimation was similarly imprecise (OR 28.6, 95% profile‐likelihood CI 1.6–523). Pooled across modalities, 97 of 250 adherent events (39%) versus 13 of 182 non‐adherent events (7%) achieved the outcome, giving an OR of 8.24 (95% CI: 4.05–16.79) with robust standard errors accounting for repeated events within patients. Restricting to recommendations made at the first visit (422 events) gave an OR of 7.98 (4.29–14.85).

Because the outcome is a global rating shared by all modalities recommended at a visit, modality‐specific estimates in patients who received several modalities cannot be attributed to a single treatment. In a post hoc analysis restricted to the 135 events at visits where only one modality was recommended, for which attribution is unambiguous, the association was preserved: 44 of 90 adherent events (49%) versus 1 of 45 non‐adherent events (2%) achieved the outcome (OR 42.1, 95% CI 5.6–318.8, *p* < 0.001), comprising 30 of 47 versus 1 of 30 for splint therapy and 10 of 34 versus 0 of 13 for professional physiotherapy (*p* = 0.043); only 11 such events involved self‐administered physiotherapy. These estimates are imprecise because non‐adherent responders were very few.

On the full seven‐level scale the proportional‐odds model gave a pooled OR of 5.79 (3.92–8.56), but the proportional‐odds assumption was formally rejected (likelihood‐ratio test against the multinomial model *χ* ^2^ = 14.53, df 3, *p* = 0.002; Brant‐type test *χ* ^2^ = 14.18, df 3, *p* = 0.003). The departure was confined to the boundary between no change and deterioration: ORs at the three improvement cut‐points were homogeneous (7.49 at PGIC ≤1, 8.24 at ≤2 and 6.53 at ≤3; test of equal coefficients *χ* ^2^ = 0.58, df 2, *p* = 0.749), whereas at PGIC ≤4 the OR was 1.28, deterioration being uncommon in both groups (12 of 250 adherent and 11 of 182 non‐adherent events). The ordinal estimate is therefore an average that understates the association with improvement and is reported as a sensitivity analysis only; the binary estimates at PGIC ≤2 and ≤3 are the principal results.

Partial adherence produced intermediate outcomes. Among patients adhering to none of their recommendations 4 of 91 (4%) achieved the outcome, among those adhering to between one third and two thirds 10 of 43 (23%), and among those adhering to more than two thirds 61 of 135 (45%); each 25‐percentage‐point increase in adherence proportion was associated with an OR of 2.00 (1.59–2.50), with a significant trend across strata (Cochran–Armitage *p* < 0.001) (Figure 2). A graded relationship of this kind is one of the classical considerations supporting a causal interpretation [33], although a continuously varying patient characteristic could produce the same pattern.

**Figure 2:**
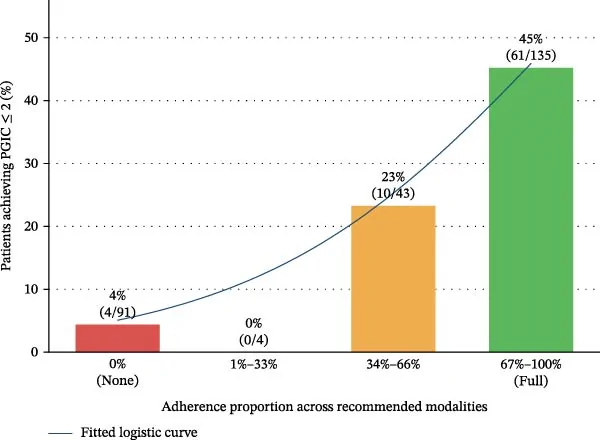
Dose–response between adherence proportion and clinically meaningful improvement (patient level, *n* = 273), with the fitted logistic curve for adherence modelled continuously.

Because discontinuation may itself be a consequence of a poor response—nine splint discontinuations were attributed to worsening pain—the exposure contrast was restricted post hoc to events never‐initiated versus events adherent, a comparison in which response‐dependent discontinuation cannot operate because non‐initiation precedes any treatment response. The association was undiminished: 8 of 123 never‐initiated events (7%) versus 97 of 250 adherent events (39%) achieved the outcome (OR 9.11, 95% CI 4.26–19.50, *p* < 0.001; splint OR 12.40, 3.57–43.00; professional physiotherapy OR 5.20, 1.89–14.29). Discontinued events had an outcome rate of 8%, close to the 7% seen in never‐initiated events. This contrast removes reverse causation through discontinuation but not the healthy‐adherer effect since patients who never start a treatment may differ systematically from those who do.

### 3.6. Baseline Predictors

Across 25 baseline characteristics screened at the patient level, only adherence and diagnostic pattern were associated with the outcome after adjustment (Table 6, Figure 3). Full adherence carried an aOR of 12.00 (5.40–26.67), essentially unchanged from its univariable OR of 9.64, indicating little confounding by the measured baseline characteristics; unmeasured characteristics may still confound the association. Joint‐only disease predicted a better response (aOR 2.80, 1.28–6.13). Bruxism showed a non‐significant trend (aOR 1.94, *p* = 0.107). Age, sex, symptom duration, psychiatric comorbidity, other chronic pain, prior splint therapy and current splint use were all unassociated. Model performance was satisfactory: *C*‐statistic 0.806, McFadden pseudo‐*R* ^2^ 0.216, Hosmer–Lemeshow *p* = 0.858, maximum variance inflation factor 2.55, with 71 events across 11 parameters. Because this corresponds to 6.5 events per variable, the model was refitted using Firth penalised likelihood; estimates were modestly attenuated but unchanged in direction or inference (full adherence aOR 10.35, 95% CI 4.83–22.20; joint‐only diagnosis aOR 2.63, 1.22–5.68).

**Figure 3:**
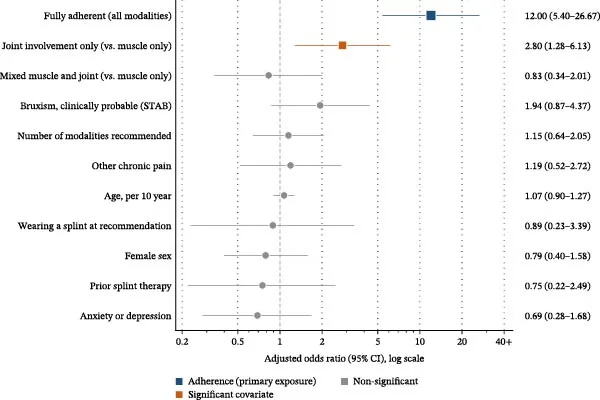
Patient‐level multivariable model for clinically meaningful improvement. Squares denote adherence and significant covariates; circles denote non‐significant covariates. Bars are 95% confidence intervals.

**Table 6:** Patient‐level multivariable logistic regression for clinically meaningful improvement (*n* = 262, 71 events).

Covariate	aOR (95% CI)	*p*
Fully adherent to all recommended modalities	12.00 (5.40–26.67)	<0.001
Joint involvement only (vs. muscle only)	2.80 (1.28–6.13)	0.010
Bruxism, clinically probable	1.94 (0.87–4.37)	0.107
Number of modalities recommended	1.15 (0.64–2.05)	0.644
Other chronic pain	1.19 (0.52–2.72)	0.683
Age, per 10 years	1.07 (0.90–1.27)	0.415
Wearing a splint at recommendation	0.89 (0.23–3.39)	0.861
Mixed muscle and joint (vs. muscle only)	0.83 (0.34–2.01)	0.676
Female sex	0.79 (0.40–1.58)	0.510
Prior splint therapy	0.75 (0.22–2.49)	0.633
Anxiety or depression	0.69 (0.28–1.68)	0.416

*Note: C*‐statistic 0.806; McFadden pseudo‐*R* ^2^ 0.216; Hosmer–Lemeshow *p* = 0.858; maximum variance inflation factor 2.55. Symptom duration was screened univariably (OR 0.85 per band, *p* = 0.189) and excluded, reducing precision without adding information. Firth penalised estimates: full adherence aOR 10.35 (4.83–22.20); joint‐only diagnosis aOR 2.63 (1.22–5.68).

Abbreviation: aOR, adjusted odds ratio.

The recommendation‐to‐outcome interval differed by adherence category (median 112 days, IQR 91–182, for adherent events; 126, 91–208, for never‐initiated events; and 147, 98–334, for discontinued events; Kruskal–Wallis *p* = 0.010), discontinued events having longer intervals, consistent with more opportunity for discontinuation to be observed. Adding the interval to the multivariable model left the adherence estimate unchanged (aOR 12.40, 5.41–28.43; interval aOR 1.01 per month, *p* = 0.751), as did restricting the model to intervals of 1–12 months (*n* = 240; aOR 10.95, 4.81–24.93).

Two univariable associations require comment. The recommendation of professional physiotherapy and the number of modalities recommended were both inversely associated with response and both attenuated to null after adjustment (number of modalities aOR 1.15, *p* = 0.644). These reflect confounding by indication, with clinicians referring more refractory patients for professional physiotherapy and prescribing more modalities in more severe presentations; they must not be read as evidence that professional physiotherapy is harmful since among patients referred, attending was strongly associated with improvement.

### 3.7. Diagnosis, Modality and Treatment Response

Because a symptomatic joint diagnosis is itself an indication for a stabilisation splint, diagnosis and modality were closely linked by clinical protocol: a splint was recommended to 91% of joint‐only patients but only 32% of muscle‐only patients, while professional physiotherapy was recommended to 80% of muscle‐only but 40% of joint‐only patients (both *p* < 0.001). Modality is therefore a consequence of diagnosis rather than a confounder of it, and we examined treatment response within diagnostic groups among adherent events only so that the comparison concerns treatments actually delivered. In these post hoc comparisons, splint therapy outperformed physiotherapy only in pure joint disease, where 34 of 54 adherent splint events (63%) achieved the primary outcome against 12 of 31 adherent physiotherapy events (39%) in the same diagnostic group (OR 2.69, 95% CI 1.08–6.68, *p* = 0.042). In muscle‐only presentations the two were indistinguishable (33% versus 31%, *p* = 1.000), as they were in mixed presentations (29% versus 31%, *p* = 1.000). Within splint events, a joint‐only diagnosis predicted response relative to any presentation involving muscle pain (aOR 2.94, 1.36–6.38, *p* = 0.006), whereas joint involvement accompanied by concurrent muscle pain conferred no advantage (aOR 1.62, 0.60–4.39, *p* = 0.341). The adherence effect itself did not differ significantly across diagnostic patterns (interaction *p* = 0.153). These comparisons are exploratory: the muscle‐only splint group comprised only 15 adherent events, and several diagnosis‐related contrasts were examined.

### 3.8. Documented Reasons for Non‐Adherence

A reason was documented for 117 of the 123 never‐initiated events, the field having been left blank in the remaining six, and for all 59 discontinuations. The entry was, however, frequently no reason given in 71 of 117 non‐initiation records (61%) and 17 of 59 discontinuation records (29%); blank fields and no‐reason‐given entries were treated alike as the absence of a substantive reason, and the following therefore describes the documented subset only and is reported as descriptive (Figure 4). The two failure pathways had almost non‐overlapping profiles. Non‐initiation was dominated by structural access barriers (26 events, 22% of documented non‐initiation records and 57% of those with a substantive reason). The single largest named barrier in the dataset was the unavailability of a physiotherapy appointment, recorded in 20 of the 66 professional physiotherapy non‐initiations (30%). Discontinuation was dominated by clinical tolerability (41 of 59, 69%); for splints specifically, inability to adjust to the appliance (17) and worsening pain (9) together accounted for 26 of the 40 splint discontinuations (65%) and only one discontinuation in the entire cohort was attributed to forgetting. Ten non‐initiation events reflected treatment not being required, a serviceable splint being already in use, another intervention planned, or symptoms having resolved.

**Figure 4:**
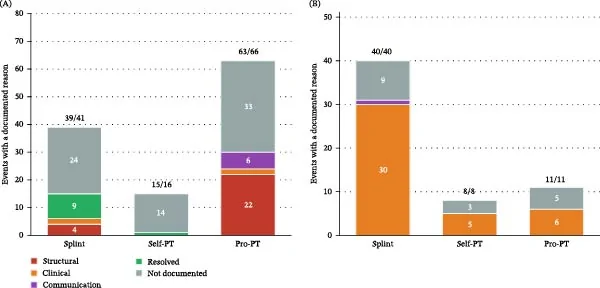
Documented barriers by failure pathway and modality. Bar labels give the number of events with a documented reason over the total number of events in that pathway; the reason field was left entirely blank in 6 of 123 never‐initiated events (splint 2, self‐administered physiotherapy 1, professional physiotherapy 3) and in none of the discontinued events. Events in which the abstractor actively recorded that no reason was given are shown in grey and form the largest single category among non‐initiations. Blank fields and no‐reason‐given entries were both treated as the absence of a substantive reason and were not distinguished in any analysis; they are shown separately for transparency only. (A) Never initiated 117 of 123 events. (B) Initiated then discontinued 59 of 59 events.

### 3.9. Secondary Analyses

In a post hoc comparison, splints fabricated within the unit were associated with a higher response rate than those obtained in the community (39 of 89, 44%, versus 11 of 44, 25%; OR 2.34, 95% CI 1.05–5.21, Fisher exact *p* = 0.038), a difference not explained by adherence, which did not differ by source, and was essentially unchanged after adjustment for it. The interval excludes unity only marginally, and this was one of several secondary comparisons, so the finding is reported as exploratory, but it converges with the prescription‐fidelity result. Pre‐treatment maximum interincisal opening was unassociated with the outcome.

## 4. Discussion

In 273 patients contributing 443 conservative treatment recommendations, roughly four in 10 recommendations were not followed, and non‐adherence was associated with a large reduction in the probability of meaningful improvement. Three findings warrant attention: the two dominant modalities differ in where in the care pathway they are lost; a specified appliance is often not the appliance received when fabrication is outsourced; and a proxy of a standardised patient‐reported outcome can be derived from routine clinical narrative in an internally consistent and auditable way.

The most actionable finding is structural. Splint therapy is distinguished by losses after the appliance is obtained: three‐quarters acquire a splint, but nearly a third of those stop using it, a discontinuation rate about three times that seen with physiotherapy, and two‐thirds of documented reasons are tolerability, principally the inability to adjust to the appliance or worsening pain. Professional physiotherapy loses patients mostly before treatment starts: two‐fifths never attend, and the largest single named barrier in the dataset is the unavailability of an appointment. These are not two expressions of a single trait called poor adherence. One is a device tolerance and titration problem, addressable by review appointments, adjustment protocols and realistic expectation‐setting at delivery; the other is a health services access problem, addressable by referral pathways, appointment capacity and funding. The distinction corresponds to the separation between initiation and persistence in the ABC taxonomy [11], and interventions aimed at patient motivation would be poorly targeted at either. Splint persistence among acquirers in our cohort (70%) is close to the two‐thirds reporting high adherence four to 8 weeks after insertion in a multicentre questionnaire study, in which perceived treatment benefit was among the strongest correlates of adherence [15]; that observation is a reminder that persistence may follow response as well as precede it.

The within‐patient comparison of the two physiotherapy modalities supports this directly. Among 66 patients recommended both, 20 performed the home programme but never attended physiotherapy, while only one did the reverse. Since these are the same individuals, the difference is unlikely to be explained by stable characteristics such as motivation, beliefs or severity; what distinguishes the two is that one requires an external appointment and the other does not, although factors specific to the referral itself, such as cost or travel, may also contribute.

Because every splint recommendation was for the same appliance, we could measure whether patients received what was prescribed. Most did, but deviation was concentrated almost entirely in community‐fabricated appliances, where the odds of receiving a different device were roughly sixfold higher, and every soft appliance came from outside the unit. This matters because soft and vacuum‐formed appliances are not equivalent to a rigid stabilisation splint and have been reported to be unhelpful or to aggravate symptoms [16, 17]. Our data cannot adjudicate that question since only 11 patients received a non‐prescribed appliance. The defensible finding is a process one that a recommendation for a specific appliance is materially less likely to be honoured when fabrication occurs outside the recommending unit, and it suggests that specifying the appliance in the referral and verifying it at review are worthwhile. The convergent signal that in‐house splints were associated with better outcomes independently of adherence is consistent with the same mechanism, although the finding was only nominally significant and arose from post hoc analysis.

The observation that splint therapy outperformed physiotherapy only in pure joint disease is consistent with the clinical rationale for the appliance and, to our knowledge, has not previously been quantified in a routine care cohort. Its converse is equally notable: adding masticatory muscle pain to a joint diagnosis abolished the advantage entirely, adherent splint response falling from 63% in joint‐only presentations to 29% in mixed ones, close to the 33% seen in muscle‐only disease. In muscle‐only disease, adherent splint and physiotherapy events responded similarly, in keeping with systematic reviews that find low‐certainty evidence for stabilisation splints in myogenous TMD and no consistent advantage over other conservative treatments [34, 35] and with a recent randomised trial in which a relaxation‐based behavioural programme was at least as effective as a splint for masticatory myalgia [36]. A stabilisation splint appears to perform best in the indication for which it is prescribed, and concurrent muscle pain attenuates that benefit. Because diagnosis determines modality under our treatment protocol, these estimates cannot be disentangled from treatment allocation and should be regarded as hypothesis‐generating, particularly given the small muscle‐only splint subgroup.

The principal methodological contribution is the derivation of a proxy of a standardised patient‐reported outcome from routine clinical narrative. The problem this addresses is general: retrospective studies of routine care are frequently constrained not by sample size but by the absence of a usable outcome, and the usual responses are to substitute a clinician‐assigned category, which is not a patient‐reported outcome, or to abandon the question. The PGIC lends itself to retrospective derivation because its response options are plain‐language descriptions rather than numerical anchors requiring calibration [20, 21].

The near‐perfect concordance with the independent clinician rating shows that the derivation is internally consistent with what a clinician recorded contemporaneously and can be audited against it. It does not establish reproducibility between assessors because a single assessor derived every score nor criterion validity against a prospectively administered PGIC, which no retrospective design can provide. The derived score is a proxy filtered through the clinician’s documentation, and the method should be understood as producing a standardised, auditable representation of what was documented rather than a substitute for administering the instrument. Prospective validation, administering the PGIC concurrently with routine documentation in a consecutive series and with duplicate blinded derivation, would establish whether the derivation systematically compresses, exaggerates or shifts the distribution.

Across 25 baseline characteristics, none predicted response after adjustment except the diagnostic pattern. Adherence, by contrast, carried an aOR of 12, was consistent across improvement thresholds, and showed a monotonic dose–response. If even part of this association is causal, effort invested in supporting adherence may yield more outcome gain than further refinement of patient selection; the design cannot establish how large that part is. We deliberately refrain from expressing the absolute differences as a number needed to treat: that metric presupposes a randomised comparison of an assigned intervention, whereas adherence here is a non‐randomised, patient‐determined exposure measured after the treatment decision, and inverting the contrast into an apparent treatment yield would imply a causal effect the design cannot support [37].

The association between adherence and improvement should therefore not be read as a treatment effect. Two mechanisms other than treatment could produce it. The first is the healthy‐adherer effect: patients who follow recommendations differ from those who do not in health behaviours, expectations, coping and other characteristics associated with a good prognosis, so that adherence predicts favourable outcomes even for inert treatments, as shown by the association between adherence to placebo and mortality in randomised trials [38, 39]. Our covariates capture only part of this, and the within‐patient physiotherapy comparison concerns initiation, not outcome. The second is response‐dependent persistence: patients who improve early may continue treatment, and those who do not may stop [15]. The never‐initiated contrast removes this second pathway for discontinuation but not the first, and the expectation of benefit or early spontaneous improvement could still influence initiation. Confounding need not act only in one direction since in one TMD cohort, patients with greater initial pain were the more compliant [14]. The ORs reported here are therefore estimates of association, not of the causal effect of adherence, which could be considerably smaller.

Several further limitations qualify these findings. The design is retrospective and observational, and adherence is not randomised. The outcome is derived rather than administered: it was reconstructed retrospectively by a single assessor who was not blinded to adherence because both were abstracted from the same narrative, and inter‐rater reliability could not be assessed. Any correlated measurement error arising from knowledge of adherence would tend to exaggerate the association. Post‐treatment pain intensity was not recorded and could not be recovered, so no continuous outcome or minimal clinically important difference analysis is available and every result rests on a single global impression item. Because one global outcome was recorded per visit and applied to every modality recommended at it, improvement in patients receiving several modalities cannot be attributed to any one of them; clustering was handled statistically, but attribution is unambiguous only in the single‐modality analysis, which was small.

Selection is a further concern. The cohort was drawn from patients with at least two visits, a criterion built into the data query, so patients seen only once, plausibly including the least engaged, could not be counted or characterised. Within the screened sample, 212 records were excluded for lack of a follow‐up appointment and 117 for insufficient narrative. If these patients were less adherent, as seems likely, the adherence rates reported here overstate those in the source population. The effect on the adherence–outcome association is less predictable: inclusion required a documented follow‐up, which may depend both on engagement and on clinical course since patients who recovered early and those who failed treatment and sought care elsewhere may not have returned. Selection on a common consequence of adherence and outcome can bias the association in either direction [40].

Reasons for non‐adherence were frequently recorded, as no reason was given. Initiation status could not be determined for 11 events, principally in the self‐administered physiotherapy group. The outcome was assessed at the first follow‐up visit after each recommendation, a single time point that varied between patients; although adjustment for the interval did not alter the association, discontinued events had longer intervals and six patients had a second assessment recorded without a new recommendation, four of whom had improved between assessments, raising the possibility that a single early assessment understates eventual response and arguing for a prespecified assessment interval in prospective work. Finally, this is a single specialist unit, and both the adherence rates and the barrier profile, particularly the prominence of physiotherapy appointment availability, are likely to reflect the local service configuration and funding arrangements.

## 5. Conclusions

Approximately 40% of conservative treatment recommendations in this TMD cohort were not followed, and non‐adherence was associated with a substantially reduced probability of clinically meaningful improvement across all three modalities, with a clear dose–response relationship; the association was undiminished when the exposure was restricted to the contrast between never‐initiated and adherent events, although residual confounding, including a healthy‐adherer effect, means that it should not be interpreted as a causal treatment effect. Non‐initiation and discontinuation are distinct failures requiring distinct remedies: access and referral capacity for physiotherapy and appliance fit and review for splints. Where splint fabrication is outsourced, the appliance received is materially less likely to be the appliance prescribed. Narrative‐derived PGIC is a feasible and internally consistent proxy for a patient‐reported global outcome in routine clinical records; it warrants prospective validation against a patient‐administered PGIC before it can be regarded as equivalent to one.

## Author Contributions

**Nadav Grinberg**: conceptualisation, methodology, investigation, writing – original draft, writing – review and editing, project administration. **Ofek Shpigler**: investigation, data curation, formal analysis (outcome derivation), writing – review and editing. **Oren Peleg, Clariel Ianculovichi and Shlomi Kleinmann**: investigation, writing – review and editing. **Reema Mahmoud**: methodology, writing – review and editing. **Ayelet Zlotogorski-Hurvitz**: resources, supervision, writing – review and editing. **Gilad Wasserman**: conceptualisation, methodology, formal analysis, validation, writing – original draft, writing – review and editing, supervision. All authors have read and approved the final manuscript.

## Funding

This research received no specific grant from any funding agency in the public, commercial or not‐for‐profit sectors.

## Disclosure

This work was part of the DMD thesis of Ofek Shpigler. The authors checked the analyses against the source data, reviewed and edited all content and take full responsibility for the content of the publication. All authors have read and approved the final manuscript.

## Conflicts of Interest

The authors declare no conflicts of interest.

## Data Availability

Data are available upon request due to privacy/ethical restrictions.
